# Epithelial-to-endothelial transition and cancer stem cells: two cornerstones of vasculogenic mimicry in malignant tumors

**DOI:** 10.18632/oncotarget.8461

**Published:** 2016-03-29

**Authors:** Baocun Sun, Danfang Zhang, Nan Zhao, Xiulan Zhao

**Affiliations:** ^1^ Department of Pathology, Tianjin Medical University, Tianjin, China; ^2^ Department of Pathology, General Hospital of Tianjin Medical University, Tianjin, China; ^3^ Department of Pathology, Cancer Hospital of Tianjin Medical University, Tianjin, China

**Keywords:** vasculogenic mimicry, epithelial-to-endothelial transition, cancer stem cells, hypoxia, LPPCN

## Abstract

Vasculogenic mimicry (VM) is a functional microcirculation pattern in malignant tumors accompanied by endothelium-dependent vessels and mosaic vessels. VM has been identified in more than 15 solid tumor types and is associated with poor differentiation, late clinical stage and poor prognosis. Classic anti-angiogenic agents do not target endothelium-dependent vessels and are not efficacious against tumors exhibiting VM. Further insight into the molecular signaling that triggers and promotes VM formation could improve anti-angiogenic therapeutics. Recent studies have shown that cancer stem cells (CSCs) and epithelium-to-endothelium transition (EET), a subtype of epithelial-to-mesenchymal transition (EMT), accelerate VM formation by stimulating tumor cell plasticity, remodeling the extracellular matrix (ECM) and connecting VM channels with host blood vessels. VM channel-lining cells originate from CSCs due to expression of EMT inducers such as Twist1, which promote EET and ECM remodeling. Hypoxia and high interstitial fluid pressure in the tumor microenvironment induce a specific type of cell death, linearly patterned programmed cell necrosis (LPPCN), which spatially guides VM and endothelium-dependent vessel networks. This review focuses on the roles of CSCs and EET in VM, and on possible novel anti-angiogenic strategies against alternative tumor vascularization.

## INTRODUCTION

Tumor growth and metastasis depends on the development of the tumor's own vascular network [1]. Avascular tumors larger than approximately 1 - 2 mm^3^ cannot receive adequate supplies of oxygen, nutrients and host-derived signaling molecules if tumor and host blood vessels are not connected [1]. However, tumor angiogenesis is complex and tumors cannot depend entirely on host endothelial cells for blood vessel formation; thus several tumor angiogenesis pathways exist, including vasculogenic mimicry (VM) and mosaic vessels [2–3].

VM was first reported by Maniotis *et al*. in 1999, who described the ability of highly aggressive melanoma cells to dedifferentiate into multiple cellular phenotypes, including those with endothelial-like characteristics that could form vessel-like structures to provide blood supply [2]. VM networks are functional vascular-like structures that carry plasma and red blood cells from host blood vessels [2, 4]. Red blood cells are found in VM channels, with no necrosis or hemorrhage around the channels [2, 5]. Fluorescent dye and activated carbon particles injected into host blood vessels were later detected in VM channels by laser scanning confocal microscopy and light microscopy [2, 4]. VM channels are lined with periodic acid-Schiff (PAS) reagent-positive extracellular matrix (ECM) and tumor cells, and endothelial cells and endothelium markers that were not identified in these structures by light microscopy, transmission electron microscopy or immunohistochemistry [2, 6]. The PAS-positive ECM also stains positive for laminin, collagens IV and VI, mucopolysaccharide and heparin sulfate glucoprotein (HSPG) [2].

Since its initial identification in human cutaneous and uveal melanomas, VM has been observedin most malignant tumors including hepatocellular carcinoma (HCC), gastric adenocarcinoma, squamous carcinoma, renal cell carcinoma, gastrointestinal stromal tumors (GISTs), tumors of the breast, ovary and prostate and most sarcomas [7–17]. VM has been widely associated with poor prognosis, large tumor volume, poor histological type, higher clinical stage and increased tumor metastasis rate or recurrence frequency [6, 18–20]. The 5-year survival rate of melanoma patients with VM is nearly 0% [14]. Our research has associated VM with poor prognosis in mesothelial sarcomas, rhabdomyosarcomas and melanomas. Triple-negative breast cancer (TNBC), the breast cancer subtype with the most pessimistic survival rates, exhibits increased VM as compared to other subtypes [19]. Sun, *et al*. reported that 88.9% of VM-positive HCC cases, but only about 50% of VM-negative cases, were at clinical stages III and IV [10]. Moreover, 72.2% of VM-positive cases suffered from metastasis or recurrence—significantly more than for VM-negative cases. Conventional anti-angiogenic agents, such as TNP-470, angiostatin and endostatin [21], have little effect on VM-positive tumors due to the absence of endothelial cells. Sunitinib, an inhibitor of VEGF receptor tyrosine kinase activity, accelerated recurrence and visceral metastasis of VM-positiveTNBC in an animal model [19].

Previous studies showed VM channel formation to involve plasticity of highly malignant tumor cells [22], ECM remodeling [3, 23] and connection of VM channels to host blood vessels [2–4]. VM channels, mosaic blood vessels and endothelial vessels coexist in malignant tumors and can switch morphologies depending on the tumor microenvironment [19]. Increasing evidence indicates that cancer stem cells (CSCs) and epithelial-to-mesenchymal transition (EMT) are essential in the formation of tumor vasculature [8, 10, 16]. This review focuses on the mechanisms by which tumor cells differentiate into endothelial-like cells and on possible therapeutic strategies to combat these alternative tumor vascularization mechanisms.

## VM AND CANCER STEM CELLS

### VM-initiating cells are of particular interest to investigators

VM tissues are PAS+/CD31−/CD34−, and do not respond to Ulex europaeus agglutinin-1 by immunohistochemistry [2]. Cells that line VM channels maintainsome characteristics of the malignant tumor, but also have some endothelial cell functions and phenotypes [2]. VM apparently mimics embryonic angiogenesis [3, 24]. Embryo vascular networks originate from mesodermal progenitor cells [25]. Angioblasts in blood islands differentiate in situ into endothelial cells and establish an original vessel network in the process of vasculogenesis [25]. In turn, the vasculogenic structure is remodeled to a mature microcirculation system from which endothelial cells proliferate to form new capillaries, in the process of angiogenesis [25].

Previous studies used microarrays to characterize the molecular signature of VM-positive melanoma tumor cells. Bittner,*et al*. and Seftor, *et al*. analyzed differentially expressed genes in highly aggressive versusless-aggressive melanomas and identified multiple phenotype-specific genes expressed in the VM-positive cells [22, 26–27]. Aside from melanoma cell-specific genes, genes particular to endothelial cells, epithelial cells, fibroblasts, hematopoietic cells, kidney cells, neuronal tissue, muscle cells and several precursor cell types were found to be expressed in aggressive melanomas [26]. These results indicated that tumor cells that could form VM might revert to a pluripotent, embryonic-like phenotype or stem cell plasticity. Observations of VM in malignant tumors with bi-directional differentiation, such as synoviosarcoma, rhabdomyosarcoma, malignant mesothelioma and epithelioid sarcoma, confirmed that to form VM, tumors must be able to differentiate pluripotently [14].

We analyzed blood vessel patterns in 169 patients with breast cancer and found that more VM-positive patients were in the TNBC group than that in the non-TNBC group [19]. An analysis of the gene expression profiles of 587 patients with TNBC showed enrichment stem cell-specific markers, including ATP binding cassette subfamily A member 8(ABCA8), protein C receptor (PROCR), aldehyde dehydrogenase1 (ALDHA1), period circadian clock 1 (PER1) and ATP binding cassette subfamily B member 1 (ABCB1), as well as markers specific to mesenchymal stem cells, such asbone morphogenetic protein 2 (BMP2), endoglin, integrin subunit alpha V (ITGAV), Thy-1 cell surface antigen (THY1), and vascular cell adhesion molecule 1 (VCAM1) [28].

Recent reports indicate that CSCs directly induce VM formation in malignant tumors. Endothelium-like CSCs exhibit enhanced plasticity and can form blood vessel-like channels.

In human renal cell carcinoma, CSC markers CD133 and CD44 were correlated marginally with VM [16], and in HCC tissues, tumor cells lining VM channels expressed SOX2 and OCT4 [29]. Glioma CSCs enriched in the human glioblastoma cell line U87 formed VM channels in xenografts; they also expressed a neural precursor marker, nestin, and multiple stem cell markers, including CD133, Oct4, Nanog and Notch1 [30]. When cultured in differentiation medium, these cells expressed glial cell marker glial fibrillary acidic protein (GFAP) and neuron markers that included neuronal class III beta-tubulin and microtubule associated protein 2 [30].

The TNBC cell line MDA-MB-231 can form VM-like channels in matrigel. Hypoxia induced by CoCl_2_*in vitro* accelerated growth of CD133^+^ MDA-MB-231 cells, which formed more VM channels when reoxygenated; cells lining the new VM channels were CD133^+^[19]. Wang R,*et al*. and Lucia Ricci-Vitiani, *et al*. found that glioblastoma endothelial cells carry the same genomic alterations as tumor cells, suggesting that many tumor endothelial cells have neoplastic origins[31–32]. They also successfully induced glioblastoma stem-like cells to differentiate into functional endothelial cells [31].

The mechanism of CSC function in VM is not yet clear, although the Notch-Nodal signaling pathway has been shown to induce VM formation. The Notch family is essential to inducing stemness and maintaining cancer cells and tumor angiogenesis. Nodal belongs to the Transforming Growth Factor-β (TGF-β) superfamily. It can bind to Cripto-1 and activate anaplastic lymphoma receptor tyrosine kinase (ALK)-4/5/7 and activin receptor IIB, which promotes tumor invasion and metastasis [33]. The Notch receptor intracellular domain breaks off from activated Notch4, which canbind to recombination signaling binding protein-J and form a cytoplasmic complex. Thiscomplex relocates into the nucleus, binds to the Nodal enhancer element and induces Nodal expression [34]. Upregulation of Nodal by Notch4 promoted VM formation in an aggressive melanoma cell line, MV3 [34].

## VM AND EMT

Tumors that are capable of forming VM include both malignant tumors with bi-directional differentiation and epithelial tumors. The transition from bi-directional differentiation tumor cells to VM-lining cells occurs in only one germ layer. The transition of epithelium-derived tumor cells into cells lining VM channels involves mesoblastic cells and endoderm cells, similar to EMT [10]. In the EMT process, epithelial cells lose many of their epithelial characteristics and instead resemble mesenchymal cells, which requires complex changes in cell architecture and behavior [35]. EMT occurs during embryogenesis, wound healing and fibrosis, and is an important step in metastasis [35]. Many EMT-inducing transcription factors, including Snail, Slug, DNA damage-responsive RNA polymerase-degradation factor (dEF1), septin interacting protein 1 (SIP1), Twist1, forkhead box C2 (FOXC2) and Goosecoid, are associated with tumor invasion and metastasis [(37-40)]. EMT is characterized by changes in the expressionof cell phenotype-specific molecules in response to EMT inducers. Various epithelium-related proteins are down-regulated, including E-cadherin, plakoglobin, occludin, zonula occludins-1, α-catenin and claudins 3/4/7 [36]. Emergence of a mesenchymal-like phenotype is indicated by upregulation of proteins such as vascular endothelial (VE)-cadherin, fibronectin, cadherin-2, vimetin, alpha smooth muscle actin and nuclear catenin-1 [37].

Recently, EMT was shown to induce VM formation. VE-cadherin was one of the first molecules identified as a VM promoter in aggressive melanoma [2]. Aggressive melanomas that lack VE-cadherin are incapable of VM[2]. Expression of the EMT-inducing transcription factor, Twist1, is associated with VM in human HCC [10, 38]. When up-regulated in human HCC HepG2 cell nuclei, Twist1 can promote VE-cadherin transcription, promoting formation of VM channels. This is accompanied by upregulation of VE-cadherin and vimentin, and downregulation of E-cadherin[10]. Similarly, Twist1 overexpression induces EMT in MCF-7 breast cancer cells, which can then form VM channels in 3D culture medium [19]. Conversely, Twist1 knockdown in MDA-MB-231 breast cancer cells impairs VM [19]. Zinc finger E-box binding homeobox 1 (ZEB1) and Slug promoted VM formation in colorectal carcinoma and HCC, respectively, by inducing EMT [39]. Wnt3a expression reduced E-cadherin levels and increased vimentin and ß-catenin expression in colon cancer. Results of *in vitro* and *in vivo* experiments showed that Wnt3a overexpression enhanced invasion and VM. Dickkopf-1, an antagonist of the Wnt/ß-catenin signaling pathway, partially reversed expression of EMT-associated proteins and VM in Wnt3a-overexpressing cells [40]. Conversely, Wnt5a suppressed colon cancer invasion by inhibiting EMT and VM [41].

During VM channel formation, epithelium-derived tumor cells differentiateinto specific cell types with some endothelial cell markers, such as VE-cadherin and vimentin. We named this process epithelial-to-endothelial transition (EET) in VM formation. EET promoted by EMT-associated inducers crosses two dermal planes, endoderm and mesoderm, and is regulated by precise mechanisms in the tumor microenvironment. Hypoxia, tumor interstitial fluid pressure (IFP) and ECM remodeling promote this process.

## PROMOTERS OF EET

### Hypoxia in the tumor microenvironment

Localized hypoxia in varying degrees is common as tumors develop, and can induce tumor aggression and metastasis by triggering angiogenesis, EMT, genomic instability, apoptotic disorders and increased CSCs [10, 42–43]. Hypoxia is involved in VM formation through several signaling pathways, and is the most potent factor affecting VM [10, 19]. Decreased oxygen levels inhibit the degradation of hypoxia-inducing factors (HIF), allowing for HIF-1α or HIF-2α nuclear localization and subsequent binding to the hypoxia-response elements of target genes, such as vascular endothelial growth factor (VEGF), VEGF receptors, EMT inducers and stemness-associated genes [44–46]. Under artificial hypoxic conditions induced by blocking a mouse femoral artery, VM channel formation was accelerated in melanoma grafts[47]. Hypoxia also induced VM formation in HCC, breast cancer, ovarian carcinoma, glioblastoma and Ewing sarcoma, both *in vivo* and *in vitro* [9–10, 19, 48–49].

Hypoxia has been linked to EET and CSC generation in VM formation. We found that activation of the EMT factor Twist1 was hypoxia-dependent in HCC [10]. Under hypoxic conditions induced by CoCl_2_, Twist1 translocates to the nucleus and binds to the VE-cadherin promoter to induce EET and VM channel formation [10]. Similarly, hypoxia-associated Twist1 overexpression upregulates VE-Cadherin in MDA-MB-231 TNBC cells, and induces these cells to generate more CSCs to promote VM channels in matrigel [19]. Notch4 is also activated by hypoxia in VM formation. HIF-1α can bind to the Notch receptor intracellular domain to stabilize the protein, promoting transcription of Nodal and contributing to VM formation [34].

### High interstitial fluid pressure and LPPCN

High interstitial fluid pressure (IFP) in the tumor microenvironment induces both endothelium-dependent vessel formation and VM. Increased IFP caused by rapid cell proliferation and disorganized perfusion in the tumor microcirculation is a barrier to host endothelial cells entering the tumor, resulting in hypoxia at the tumor center [50]. This hypoxia triggers stem-like tumor cells to form VM channels and connect to endothelium-dependent vessels [19]. Mouse melanoma B16 cells were grafted into microenvironments with varying pressures [51]. Melanomas in hind limb skeletal muscle under high pressures were more aggressive than those in the abdominal cavity at low pressures [51]. High microenvironmental pressures increased MMP-2 and MMP-9 expression in melanoma cells, induced ECM remodeling and VM channel formation and accelerated tumor cell invasion [51]. Although GISTs are typically not highly aggressive, VM was identified in 21 of 84 GIST specimens in which MMP-2 and MMP-9 were highly expressed, but endothelium-dependent vessels were rare [13]. GISTs are thought to originate from Cajal's cells in mesodermal tissue or from multipotent mesenchymal stem cells [52]. High interstitial pressures in GISTs as a result of dense tissue limit growth of endothelium-dependent vessels; the resulting hypoxia facilitates tumor stem-like cell transdifferentiation and VM.

Although VM can occur in a tumor microenvironment with high IFP, the space requirements for VM formation are unclear. Some networks of darkly stained tumor cells were observed when VM was initiated in a very small melanoma [53]. These networks invariably accompanied VM channels and were even connected to VM channels [53]. When Zhao and Han, *et al*. used a computer image analysis and a computer 3D rebuilding technique to analyze the relation of these cells and microvessels depended on tumor growth, they found that the peak value of these special cells appearance was followed by the peak value of VM channels formation in time line in a B16 melanoma-bearing mouse model [54] (unpublished data). The correlation between VM network and these cells in space and time suggests that these cells are pioneers in VM formation. Immunohistochemistry, transferase-mediated deoxyuridine triphosphate-biotin nick end labeling (TUNEL) staining and electron microscopy revealed that these cells exhibited a necrotic morphology, but expressed some apoptosis-associated proteins [53]. We coined the term “linearly patterned programmed cell necrosis” (LPPCN) to describe them [53]. LPPCN and VM coexistence predicts poor prognosis in human melanoma patients. Furthermore, high IFP and hypoxia were shown to promote LPPCN and VM channels. Tumor cells undergoing LPPCN under conditions of high IFP and hypoxia expressed increased Bax, Bcl-2, cytochrome c, caspase3, caspase9, calpain1, caspase8 and Fas. Inhibitors of caspase 3, caspase 8 and caspase 9 also inhibited LPPCN and VM (unpublished data).

In HCC, the proapoptotic factor bcl-2 is positively correlated with VM and with nuclear expression of EMT-regulating transcription factor Twist1 [55]. Hypoxia induces Bcl-2 and Twist1 coexpression in HCC HpG2 cell nuclei, and co-immunoprecipitation and knockdown experiments found that two separate Bcl-2 domains bound to a Twist1helix-loop-helix DNA binding domain [55–56]. The Bcl-2/Twist1 complex facilitates Twist1 nuclear transport and leads to transcriptional activation of a wide range of genes that induce tumor cell EET and VM [55]. Hence, LPPCN not only provides the spatial basis for VM channels, but also promotes VM through the interaction of LPPCN-related proteins and EMT factors.

### ECM remodeling

ECM remodeling is a critical step in VM channel formation and angiogenesis. A PAS-positive ECM layer coats the internal surfaces of VM channels. Known PAS-positive substances include laminin, collagens IV and VI, mucopolysaccharide, HSPG and F-tissue factor (TF), TF pathway inhibitor 1 (TFPI-1) and 2 (TFPI-2) [2, 57]. Blood vessel basement membranes also include laminin, collagens IV and VI, and mucopolysaccharide [2]. Hence, ECM remodeling is needed for both VM channel construction and to facilitate connection of VM and endothelium-dependent vessels [2, 58]. A balance of TF and TFPI-1 is thought to modulate anticoagulant functions to maintain flow in VM channels [57].

Matrix metalloproteases (MMPs) have important functions in ECM remodeling for VM channel formation. In melanoma, both MMP-14 and MMP-2 degrade laminin-5γ2-chain into promigratory γ2′ and γ2x fragments, which then stimulate invasion and VM formation [59]. MMP-2 and MMP-9 are linked to VM and poor prognosis in cancers of the breast, ovaries, prostate, liver, stomach and kidneys, along with GISTs, mesothelial sarcomas, rhabdomyosarcomas and melanomas [6, 9–10, 13–14, 16]. Recently, ECM remodeling in VM was shown to be a chain reaction of EET. Overexpression of EMT transcription factor Twist1 under hypoxic conditions increased VE-cadherin, MMP-2 and MMP-9 expression and VM formation in HCC cells [10]. Twist1 is positively correlated with VE-cadherin, MMP-2, and MMP-9 expression in VM-positive human HCC [10]. VE-cadherin is an endothelium marker and a promoter of ECM remodeling in VM, and can phosphorylate erythropoietin-producing HCC-A2 (EphA2), activating PI3K to promote MMP-14 and MMP-2. Activated MMP-14 and MMP-2 degrade ECM to facilitate VM channel formation [24, 59].

Recently, Zhao,*et al*. found that collagenase-3 (MMP-13) specifically inhibits ECM remodeling in VM. Unlike MMP-2, -9 and -14, MMP-13 inhibits VM in melanoma. MMP-2 and MMP-14 degrade laminin-5 into 105-kDa laminin-5 γ2′ and 80-kDa laminin-5 γ2x, which MMP-13 then cleaves into ~40-kDa fragments that are not used in VM [60]. MMP-13 also cleaves VE-cadherin to bind to β-catenin, forming a complex that inhibits β-catenin translocation, thus inhibiting VM and melanoma invasion [60].

## TRANSLATIONAL SIGNIFICANCE OF EET AND CSCS IN VM

In malignant tumors, VM is a marker for poor prognosis and presents a challenge to traditional anti-angiogenic treatments. Most anti-angiogenic agents in clinics and in trials target endothelium-dependent vessels by blocking VEGF, VEFGRs, epidermal growth factor receptor (EGFR), or platelet-derived growth factor receptors (PDGFRs) [61]. These anti-angiogenic agents cannot inhibit tumor growth in many individual patients, and in some cases can promote tumor invasion and metastasis [61–62]. The precise reasons for treatment failure with these anti-angiogenic agents are not clear. The mechanisms of VM formation differ from those of endothelium-dependent vessels; VM channels also expose tumor cells directly to blood flow, which facilitates metastasis and may reduce the efficacy of conventional anti-angiogenic agents in VM-positive cases. For example, TNBCs that had been treated with sunitinib regrew and metastasized after sunitinib administration was terminated, as sunitinib treatment induced hypoxia, leading to formation of new VM channels [19]. After discontinuing treatment, endothelial vessels rebounded and linked to these VM channels [19]. The angiogenesis inhibitor, endostatin (an integrin signaling inhibitor), also failed to suppress VM channels formed by human melanoma MUM-2B and C8161 cells *in vitro* [21]. VM must be considered when devising strategies that utilize standard VEGF-targeted therapies or endothelium-dependent drugs.

Thalidomide is the first drug that has been proved to inhibit VM formation in mouse melanoma [63]. Thalidomide is a teratogenic sedative and a reactive oxygen species producer that targets TNF-α. It can reduce expression of MMP-2 and MMP-9. In addition to blocking ECM remodeling, thalidomide also inhibits VM by interfering with anti-apoptotic, pro-angiogenesis and pro-metastatic functions of the NF-κB pathway [63]. Other drugs that have been shown to inhibit VM include the mTOR-specific inhibitor rapamycin, which reduced VM channels formed by malignant U87glioblastoma cells by inhibiting MMPs and HIF-1α[64]. Genistein and curcumin, both botanical extracts, can inhibit VE-cadherin → EphA2→ MMPs cascade to inhibit VM in melanoma *in vivo* [(70-72)]. A better understanding of VM channel formation will provide additional potential molecular targets for anti-angiogenic therapies.

As CSCs and EET have been shown to promote VM in malignant tumors, molecular regulators of EET and CSCs are candidate therapeutic targets in VM-positive tumors. Doxycycline, a tetracycline derivative, prolonged survival inmice with human liver tumors by inhibiting degradation of E-cadherin, thus preventing EET and VM. *In vitro* experiments implicated methylation of the E-cadherin gene and downregulation of the EMT promoters, vimentin and VE-cadherin, in the prevention of EET and VM [65].

The Notch pathway in CSCs is another possible target for VM inhibition. Anti-Notch4 antibody impaired VM in C8161 and SK-MEL-28 melanoma cells, and diminished clonogenicity *in vitro* by downregulating Nodal expression [34]. Similarly, shTwist1 disrupted VM in MDA-MB-231 TNBC cells grown on 3D matrigel under hypoxic conditions by reducing the CSC population[19]. Moreover, dual antiplatelet therapy, a gamma-secretase inhibitor, was found to inhibit glioblastoma CSCs from differentiating into endothelial-like progenitor cells through blockade of Dll4-Notch signaling [31]. A single treatment of bevacizumab, a recombinant humanized VEGF monoclonal antibody, did not influence the function of CSCs. However, the combination of both remarkably inhibited tumor growth in a mouse model with glioblastoma and fibrosarcoma engrafts [31]. These results suggest that a combination of anti-angiogenic therapies against multiple targets would be efficacious in malignant tumors with VM.

## CONCLUSIONS

**Figure 1:**
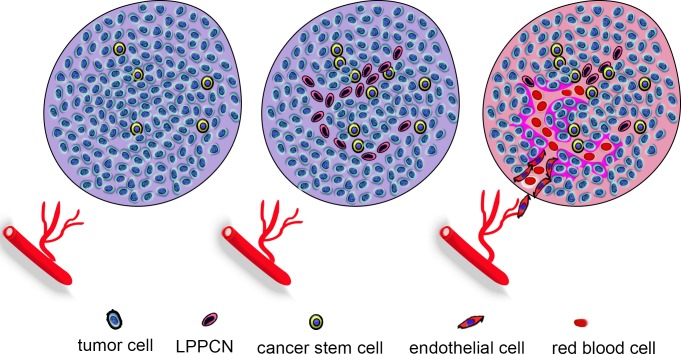
Hypoxia and IFP induce VM formation When endothelium-dependent vessels do not grow into the tumor, the resulting hypoxia promotes VM formation to provide selective perfusion, leading to tumor aggression and metastasis. CSCs and EET accelerateVM formation by inducing tumor cell plasticity, remodeling ECM, and connecting VM channels and host blood vessels. Hypoxia and high IFP in the tumor microenvironment induce LPPCN in some tumor cells, to provide a spatial guide for VM and endothelium-dependent networks. EMT-inducing transcription factors may induce CSCs to differentiate into endothelium-like VE-cadherin+ cells in order to form VM channels with ECM remodeling. These channels then connect to endothelium-dependent vessels and areperfused withblood.

The functional tumor microcirculation system includes VM, endothelium-dependent vessels and mosaic vessels. When endothelium-dependent vessels are inhibited by traditional anti-angiogenic therapies, the resulting hypoxia promotes VM formation to provide selective perfusion, leading to tumor aggression and metastasis. CSCs and EET promote VM formation by accelerating tumor cell plasticity, remodeling ECM, and connecting VM channels and host blood vessels. Hypoxia and high IFP in the tumor microenvironment induce LPPCN in some tumor cells to provide a spatial guide for VM and endothelium-dependent networks. EMT-inducing transcription factors may induce CSCs to differentiate into endothelium-like VE-cadherin+ cells in order to form VM channels with ECM remodeling. These channels can then connect to endothelium-dependent vessels and be perfused with blood (Figure 1). The molecular regulators HIF-1α, Twist1, Notch4, VE-cadherin, EphA2, MMP-2, MMP-9 and MMP-13 are potential therapeutic targets and prognostic indicators.
